# COVID-19 positive cases in a pediatric surgery department from Romania: Case series from 2 years of pandemics

**DOI:** 10.1097/MD.0000000000036235

**Published:** 2023-12-01

**Authors:** Florin Filip, Ramona Avramia, Monica Terteliu-Baitan, Maria - Elena Cocuz, Roxana Filip

**Affiliations:** a College of Medicine and Biological Sciences, Stefan cel Mare University of Suceava, Suceava, Romania; b Suceava Emergency County Hospital, Suceava, Romania; c Fundamental Prophylactic and Clinical Disciplines Department, Faculty of Medicine, Transilvania University of Brasov, Brașov, Romania; d Clinical Infectious Diseases Hospital of Brasov, Brasov, Romania.

**Keywords:** acute appendicitis, COVID-19, fracture, monocytosis, pediatric surgery, RT-PCR test, soft tissue abscess

## Abstract

**Rationale:**

The COVID-19 pandemic had a dramatic effect on various health systems in terms of admissions and outcomes, including pediatric surgery activity. The aim of this paper was to analyze the outcome of SARS-CoV-2-positive patients admitted to our department during the regional COVID-19 pandemic in North-Eastern Romania. We also evaluated the changes generated in our daily practice by the COVID-19 pandemic and the dynamic response to this major challenge.

**Patient concerns:**

The patients presented with symptoms related to their primary diagnosis: local pain and deformity in case of fractures; pain, swelling, and erythema in case of abscess; pain and decreased range of motion (ROM) in case of intolerance to metal implants. Other specific concerns are mentioned on an individual basis.

**Diagnoses:**

Eighteen patients (of which 4 had acute appendicitis and were included in a previous article), representing 1.18% of the total number of admissions, tested positive for SARS-CoV-2. There were 4 patients with fractures, 3 patients with soft tissue abscess or cellulitis, 2 patients with intolerance to metal implants, 1 patient with facial burn, 1 patient with thumb laceration, 1 patient with liver trauma, 1 patient with undescende testis, and 1 patient with symptomatic inguinal hernia, respectively. Boys represented 11/ 14 (78.57%) of the cases. The mean age of the patients was 9 years 11 months. There were only mild COVID-19 cases.

**Interventions:**

Surgery was performed in 13/ 14 (95.71%) of cases. The fractures were treated with open reduction internal fixation (ORIF); incision and drainage (I & D) were performed in case of soft tissue abscess; the metal implants were removed in case of local intolerance. Other conditions (burn, inguinal hernia, undescended testis, skin laceration) were treated specifically. Only 1 patient with liver laceration was treated conservatively under close hemodynamic monitoring.

**Outcomes:**

The mean length of stay (LoS) was 2.71 days. The infection with the SARS-CoV-2 virus had no deleterious effect on the surgical outcome among the 14 patients included in the study. There were no surgical complications during admission and no patient returned for late complications related to their primary disease or SARS-CoV-2 infection.

**Lessons:**

The SARS-CoV-2 infection had no significant influence on the outcome of pediatric surgical cases included in the study. We noticed a significant (31.54%) decrease in the number of admissions compared to the previous 2-year interval before the COVID-19 pandemic. Fast and adequate adjustment of the daily activity imposed by the COVID-19 pandemic was feasible and may be used in the future should similar epidemiological emergencies occur.

## 1. Introduction

On March 11, 2020, COVID-19 was officially declared a pandemic following the rapid transmission of infection with the SARS-CoV-2 virus in multiple countries.^[1–3]^ The pandemic had a dramatic effect on national health systems worldwide in terms of patient care, personnel availability, and clinical management.^[4,5]^ Children had a similar risk as adults to be infected with SARS-CoV-2 virus, but they were asymptomatic or developed milder forms of COVID-19 disease at the beginning of the outbreak. Most pediatric cases presented with respiratory or nonspecific symptoms, such as fever, cough, myalgia, and fatigue.^[6–9]^ Gastrointestinal symptoms (sometimes mimicking acute surgical diseases) and thrombotic complications were also recorded in COVID-19 patients, including children.^[10–13]^ It was estimated that less than 20% of the pediatric cases were severe, with nearly one-third of them requiring ICU admission. The overall case-fatality rate in children was initially calculated to be 2.3% by the Chinese Center for Disease Control and Prevention.^[6,14–16]^ This rate increased to 50% in severe cases of COVID-19. Older age and underlying comorbidities were associated with unfavorable outcomes, such as severe/ protracted disease, MSOF (Multiple System Organ Failure) requiring admission to the intensive care unit (ICU), and death.^[6,10,17]^

Several reports have estimated the impact of COVID- 19 pandemic on the usual pediatric surgical activity, including emergency cases such as, but not limited to, acute appendicitis.^[18–21]^ There has been a general perception that COVID-19 has delayed presentations for emergent surgical cases in children, or was responsible for an increased incidence of complicated surgical cases.^[22,23]^ Not all studies, however, were able to confirm this presumption.^[24]^ To address these problems, the American College of Surgeons has released guidelines stating that all elective procedures should be postponed or performed in an ambulatory surgery center if feasible.^[25]^ It was recommended that surgeries should be performed if their delay may cause harm to patients, increase the length of stay (LoS) in the hospital, or facilitate their readmission. The only exceptions were considered most of the oncological and high-emergency surgical procedures.^[26–28]^

The article is presenting the characteristics and the management of COVID-19-positive pediatric surgical patients in a Pediatric Surgery Department located in Suceava County, North-Eastern Romania. Every effort was made to ensure safe and smooth continuous care of pediatric surgical cases during the COVID-19 pandemic. Standard isolation precautions for patients and staff were taken and the hospital circuits were adapted to the new epidemiological conditions. The length of stay was reduced in order to minimize the contact between the COVID-19 patients and the non–COVID-19 patients and medical staff, respectively. By adopting the daily workflow and the clinical management to the COVID-19 epidemiological context, we were able to deliver appropriate care to our pediatric surgical patients. This experience would also allow appropriate adjustment to any future challenges raised by situations similar to the COVID-19 pandemic.

## 2. Materials and methods

We performed a retrospective study of COVID-19-positive children admitted to our department between April 1, 2020, and March 31, 2022. The methodology of the study was similar to the one reported in previous studies.^[29–31]^ Informed consent for treatment and data sharing/ publication had been obtained in all cases from the legal guardians at admission, in order to make use of these data for research purposes. Consent from the Ethical Committee of the hospital was obtained and the patients were deidentified. All patients were tested for SARS-CoV-2 infection at admission using either the RT-PCR or rapid antigen testing, according to the national COVID-19 protocol in use at that time. Only patients who underwent a surgical procedure during their admission were finally selected. The variables collected were age, sex, race/ethnicity, diagnosis, surgical procedure, LoS, the severity of SARS-CoV-2 infection, postoperative complications, pediatric intensive care unit (PICU) admission or duration of supplemental oxygen (O_2_) use. Clinical data also included X-Ray/US imaging findings and significant results of laboratory investigations performed at admission. All study data were sourced from existing hospital administrative databases or electronic medical records by the primary author. The outcome of the study was represented by variables related to surgery and admission: type of procedure, complications, length of stay, and use of O_2_. We also analyzed if there was a decrease in the number of cases admitted during and before the COVID-19 pandemic in our department. A correlation between the level of monocytosis and disease severity gravity was also searched.

According to the hospital’s policy, only emergency or urgent pediatric surgical cases in COVID-19-positive patients were selected for surgery; elective cases or other conditions in which surgery could be delayed were treated conservatively. This approach was in accordance with standard surgical guidelines released during the COVID-19 pandemic, such as those recommended by ACS and endorsed by American Pediatric Surgical Association.^[25–28]^

**Figure 1.**
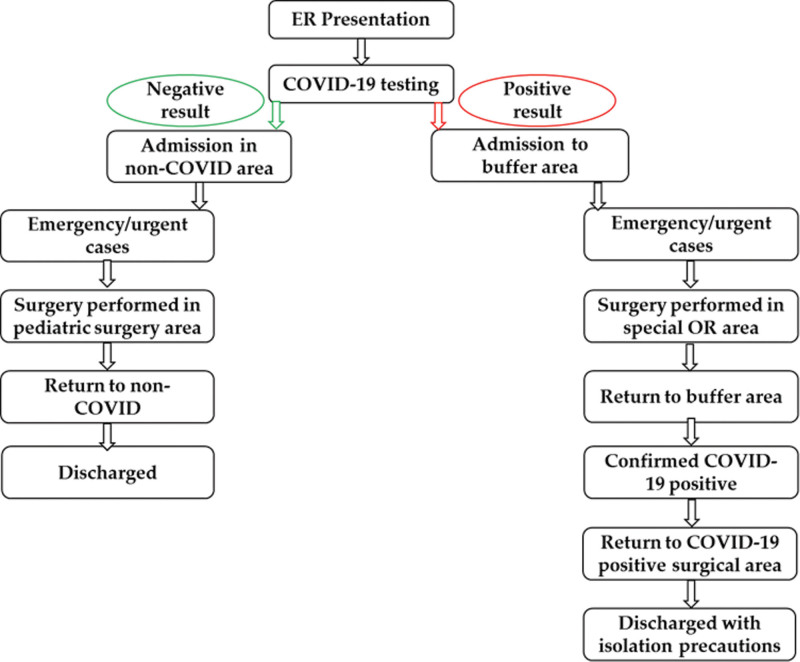
Workflow diagram of pediatric surgery patients.

All patients were initially admitted to a buffer surgical area, waiting for the result of PCR tests to become available. For patients with positive results for SARS-CoV-2 infection who required surgical treatment, surgery was performed in a COVID-19 designated operating room. After surgery, the patients who tested positive for SARS-CoV-2 infection were transferred to a special area in the hospital where standard isolation precautions were used. The patients were sent home in isolation as soon as they fulfilled standard discharge criteria in order to minimize their stay in the hospital (Fig. 1). Disease severity was classified into 3 groups, according to literature data: (1) Mild – did not require supplemental oxygen, inotropes, or PICU admission; (2) Moderate – required supplemental oxygen via nasal cannula or face mask, and may have required PICU admission, but did not need intubation or inotropic support; (3) Severe – required mechanical ventilation or inotropic support in the PICU.^[29]^

## 3. Results

Between April 1, 2020, and March 31, 2022, a number of 1.598 children were admitted to our department. This number represented a significant decrease compared to a similar time interval 2 years before (April 1, 2018–March 31, 2020), with 2.334 patients discharged (a decrease of 31.54%). Of these patients, a number of 18 (1.13%) who underwent surgery tested positive for SARS-CoV-2 infection. Four (4) patients from this group were diagnosed with acute appendicitis and they were included in a previous article.^[30]^ The main data related to the remaining 14 patients are presented in Table 1.

**Table 1:** Clinical and laboratory data for the 14 COVID-19-positive patients.

No.	Age (yr + mo)	Sex	Diagnosis	COVID-19 status	WBC at admission	Surgical procedure	Clinical course	Follow-up
1.	12 + 7	M	Displaced fracture of the distal left forearm	Mild	Total WBC = 7.900 × 10^3^ Neu = 4.760 × 10^3^/60.3% Lym = 1.790 × 10^3^/22.7% Mo = 0.99 × 10^3^/12.5% (monocytosis)	ORIF with 2 TENs	No complications, no O_2_ requirements, 3-d admission	Discharged home* with isolations precautions/treatment† for 12 d
2.	17 + 8	F	Facial thermal burn (2nd degree, 1% Body Surface Area – BSA)	Mild	Total WBC = 7.9 × 10^3^ Neu = 7.52 × 10^3^/76% Lym = 1.79 × 10^3^/22.7%	Local debridement, burn area dressing	Diagnosed with rapid Ag test No complications, no O_2_ requirements, 1-d admission. Ophthalmology and ENT consult to rule out complications	Discharged home with isolations precautions/treatment for 14 d
3.	10 + 2	F	Fracture of the right radial neck S/P ORIF. Elbow stiffness, local cellulitis	Mild	Total WBC = 6.920 × 10^3^ Neu = 3.12 × 10^3^/45. 1% Lym = 2.3 × 10^3^/33. 2%, Mo: 0. 76 × 10^3^/11% (monocytosis)	Removal of a 2.5 mm titanium elastic nail (TEN), right radius	No complications, no O_2_ requirements, 2-d admission.	Discharged home with isolations precautions/treatment for 12 d
4.	9 + 2	M	Displaced fracture of the left radial neck, 4th degree	Mild	Total WBC = 6.93 × 10^3^ Neu = 4.48 × 10^3^/64. 7%; Lym = 1.59 × 10^3^/22. 9% Mo: 0,.19 × 10^3^/8,4% (monocytosis)	ORIF with 1 TEN left radial neck	No complications, no O_2_ requirements, 2-d admission	Discharged home with isolations precautions/treatment for 12 d
5.	12 + 6	M	Displaced fracture of the middle 1/3rd left forearm	Mild	Total WBC = 6.93 × 10^3^ Neu = 4.48 × 10^3^/64. 7%; Lym = 1.59 × 10^3^/22. 9% Mo: 0.19 × 10^3^/ 8. 4% (monocytosis)	ORIF with 2 TENs	No complications, no O_2_ requirements, 2-d admission	Discharged home with isolations precautions/treatment for 12 d
6.	10 + 2	M	Left forearm cellulitis following ORIF for forearm fracture	Mild	Total WBC = 9.600 × 10^3^ Neu = 3.110 × 10^3^/32.1% Lym = 3.880 × 10^3^/40%	Removal of 2 TENs left forearm, local debridement	No complications, no O_2_ requirements, 2-d admission	Discharged home with isolations precautions/treatment for 12 d
7.	1 + 1	M	Undescended left testis. Left testicular torsion	Mild	Total WBC = 11.270 × 10^3^ Neu = 1.600 × 10^3^/14.2% Lym = 8.860 × 10^3^/78.6% (neutropenia, lymphocytosis)	Left orchiopexy	No complications, no O_2_ requirements, 4-d admission	Discharged home with isolations precautions/treatment for 10 d
8.	16	M	Liver laceration. Hemoperitoneum. Multiple rib fractures	Mild	Total WBC = 10.420 × 10^3^ Neu = 7.330 × 10^3^/70.3% Lym = 2.150 × 10^3^/20.6 % Mo = 0.83 × 10^3^/8% (lymphopenia, monocytosis)	Conservative treatment, *i.v*. hydration, bed rest, pain control, hemostatic	One (1) d ICU admission for vital signs monitoring No complications, no O_2_ requirements, 2-d admission	Discharged home with isolations precautions/treatment for 12 d
9.	6 + 5	M	Left thumb accidental trauma with disruption of the flexor muscles	Mild	Total WBC = 7, 32 × 10^3^ Neu = 2, 52 × 10^3^/34.4% Lym = 3. 77 × 10^3^/51. 5%	Flexor tendons suture, local debridement, casting	No complications, no O_2_ requirements, 4-d admission	Discharged home with isolations precautions/treatment for 10 d
10.	9 + 9	M	Left axillary abscess. Cellulitis	Mild	Total WBC = 8. 7 × 10^3^ Neu = 6 × 10^3^/69% Lym = 1. 87 × 10^3^/21.5% (neutrophilia and lymphopenia)	I & D, debridement, dressing	No complications, no O_2_ requirements, 2-d admission	Discharged home with isolations precautions/treatment for 12 d
11.	13 + 1	F	Displaced fracture, right clavicle	Mild	Total WBC = 5.81 × 10^3^ Neu = 4.35 × 10^3^/74.8% Lym = 1.17 × 10^3^/20.2% (lymphopenia)	ORIF (plate and screws)	No complications, no O_2_ requirements, 2-d admission	Discharged home with isolations precautions/treatment for 12 d
12.	17 + 5	M	Pilonidal abscess	Mild	Total WBC = 18.51 × 10^3^ Neu = 14.46 × 10^3^/78.1% Lym = 2.89 × 10^3^/15. 6 % (lymphopenia)	I & D, debridement, dressing	No complications, no O_2_ requirements, 5-d admission	Discharged home with isolations precautions/treatment for 9 d
13.	1 + 7	M	Symptomatic bilateral inguinal hernia	Mild	Total WBC = 11.73 × 10^3^; Neu = 3.96 × 10^3^/33.8% Lym = 6.18 × 10^3^/52.7% (neutropenia, lymphocytosis)	Inguinal hernia repair, bilateral	No complications, no O_2_ requirements, 2-d admission	Discharged home with isolations precautions/treatment for 12 d
14.	1	M	Perianal abscess	Mild	Total WBC = 16.22 × 10^3^ Neu = 3.94 × 10^3^/24.3%; Lym = 10.46 × 10^3^/64.5% Mo = 1.2 × 103/7.4% (neutropenia, lymphocytosis, and monocytosis)	I & D, debridement, dressing	No complications, no O_2_ requirements, 5-d admission	Discharged home with isolations precautions/treatment for 9 d

F = female, I & D = incision and drainage, Lym = lymphocyte count, M = male, Mo = monocyte count, Neu = neutrophil count, ORIF = open reduction & internal fixation, WBC = white blood cell count.

* Discharged home if proper isolation criteria met.

† Treatment generally included multi-vitamins and isoprinosin for 30 d.

There were 11 (78.57% of total cases) boys and 3 (21.43%) girls in this group of patients; the age of the patients ranged from 1 year and 17 years and 8 months, with a mean age of 9 years and 11 months. Most cases were represented by limb fractures – 4 (24.57% of the total number) cases, followed by soft tissue abscesses with cellulitis – 3 patients (21, 42% of total cases) and other common surgical conditions in children (trauma, inguinal hernia, testicular torsion, burn, and removal of metal implants which generate local cellulitis). There were no children with cardio-thoracic surgical conditions, as these patients undergo surgery in specialized centers.

The mean LoS was 2.71 days, ranging from 1 day (a 17-year-old girl with facial burn) and 5 days (a 1-year-old boy with perineal abscess and a 17-year-old boy with pilonidal abscess, respectively). Of the 14 patients, 10 (71.43%) were admitted in a relatively short time interval, from September 2020 to December 2020; the other 4 (22. 22%) were treated until April 2022. However, sporadic presentations of SARS-CoV-2 patients are still recorded and managed in a similar manner in our Pediatric Surgery Department. All patients were classified as having mild forms of COVID-19 disease, as stated above. There were no complications recorded during their hospitalization.

Analysis of white blood cell count levels showed that 6 (42.85%) patients presented monocytosis at ad-mission, of which 1 patient also had associated lymphopenia, and 1 patient had a combination of neutropenia, lymphocytosis, and monocytosis. Two (2) patients had isolated lymphopenia, another 1 patient had neutrophilia and lymphopenia, and 2 patients had neutropenia and lymphocytosis. Three (3) patients had normal levels of white blood cell count at admission.

The COVID-19 status was investigated according to the public health regulations im-plemented at the time of admission. All the patients had an RT-PCR test or a rapid antigen test taken in ER and then were admitted in a buffer area waiting for the test result to become available. Surgery was performed in a specially designated operating room (OR), where specific precautions for SARS-CoV-2 infection were available. They returned to the buffer area until the result of the test became available, at which point they were transferred to the COVID-19 area if the test were positive. The discharge criteria were similar to those used before the COVID-19 pandemic; however, all the patients received standard instructions for isolation released by the MoH in order to avoid the spread of SARS-CoV-2 infection in the population.

## 4. Discussions

Several articles demonstrated that the COVID-19 pandemic had a serious impact on pediatric surgery services.^[18,19,31–34]^ The overall influence on pediatric surgery activity, as well as the impact on the specific type of surgical procedures, including elective and outpatient services, were analyzed.^[35–37]^ In many hospitals, elective surgeries were temporarily suspended to ensure adequate hospital capacity to respond to the rapid spikes in COVID-19 cases. This approach also decreased the risk of nosocomial transmission of SARS-CoV-2 infection. Countries that implemented these policies included the USA, Australia, Finland, and other Nordic countries.^[38–41]^ Because delay of surgery for “time-sensitive” and urgent diseases in children could be dangerous for their health, some centers continued to perform elective surgical procedures for special conditions, such as neonates and oncology cases.^[42–44]^ A declining trend in neonates and oncology cases was still identified in some centers as many families were reluctant to present with their children to the hospital during the COVID-19 pandemic.^[19,45]^

Apparently, the number of emergency procedures performed on children was not affected by the COVID-19 pandemic. One study found that the number of laparotomies performed for perforated appendicitis was similar before and during the pandemic.^[31]^ In the USA, some hospitals encouraged the non-operative management of acute appendicitis, while others continued to perform routine appendectomies.^[19]^ Huang et al^[46]^ found that appendectomy had a higher success rate and reduced the length of hospital stay (LoS) compared to conservative treatment with antibiotics. In addition, about 10% of the children undergoing nonoperative treatment would eventually require appendectomy before discharge, which increased the LoS and the risk of exposure to COVID-19 among patients and medical staff. Notably, no consensus has finally been established for the management of acute appendicitis during the COVID-19 pandemic. The choice between conservative treatment and appendectomy actually depended on the resources of each institution.^[18–20]^ The operative approach decreased the LoS and allowed more beds and medical staff to become available; in some cases, same day discharge after surgery was implemented during the COVID-19 pandemic.^[46]^ The non-operative treatment, on the other side, decreases the risk of COVID-19 exposure for patients and physicians in the OR and during aerosol-producing maneuvers, such as intubation.^[47]^

Previous studies have estimated the overall incidence of COVID-19 in children undergoing preoperative universal screening to be less than 1%. Half of the patients with positive results had no symptoms.^[31]^ Symptomatology was found to be an inadequate differentiator criterion for SARS-CoV-2 infection in children.^[48]^ Even in symptomatic children, some of the clinical features could be mild or attributed to unrelated conditions (e.g., acute appendicitis or gastrointestinal disease).^[49]^ The incidence of COVID-19 in children requiring time-sensitive surgery may not represent the incidence in children undergoing elective surgery. Finally, when attempting to identify all patients with COVID-19 even if surgery was postponed owing to positive testing, cases may have been missed, resulting in an underestimation of the true incidence.^[31,49]^ The gradual return to a “new normal” pediatric surgery activity became possible after several policies started to be implemented by various local and national health authorities. In an example from a large pediatric surgery center in Indonesia,^[33]^ one major surgery started to be performed every Tuesday, while 2 or 3 minor procedures were scheduled and conducted on another working day. Furthermore, pediatric surgeons were encouraged to share their experiences and best practices achieved during the COVID-19 pandemic.^[20]^ Another recommendation included the use of universal testing for all elective or emergency procedures, according to specific factors, such as regional COVID-19 prevalence, local testing capability, and availability of personnel and protective equipment. The value of universal screening was shown to be greatest in areas with higher prevalence.^[49]^

This paper has several limitations due to its single-center retrospective nature with subsequent biases as well as the inability to collect additional variables of interest or determine cause-and-effect. It included both orthopedic and surgical cases admitted to our department. There was no intention to look forward to specific pathological conditions, but rather evaluate the influence of SARS-CoV-2- infection on patients’ outcomes and the response of our surgical team to the pandemic. The significant decrease in the volume of admissions can be reasonably attributed to the COVID-19 pandemic, as several papers have already outlined.^[42,44,46]^ Another limitation of the study is represented by the fact that it included pediatric surgical patients admitted to an emergency hospital located in a geographical area with a very high incidence of SARS-CoV-2 infection in Romania.

Our hospital primarily serves urban and rural areas of Suceava County, with an estimated population of 800,000 people. There has been an increasing number of COVID-19 cases in this area starting in March 2020, first related to the return of Romanian citizens from highly affected areas in Europe, such as Italy or France.^[50]^ Shortly thereafter, local transmission surpassed the importation of cases, with the occurrence of documented patterns of COVID-19 spread, such as: inside hospitals, at workplaces, within families, or via carriers in closed public spaces.^[51–53]^ On March 26^th^, 2020, the total number of confirmed cases in Romania exceeded 1000. An increasing number of Romanian hospitals, including Suceava County Hospital, were designated by the Romanian Ministry of Health (MoH) as referral units for patients with SARS-CoV-2 infection.^[54]^ As infections started to be reported among medical personnel, many wards were closed and patients with other conditions were transferred within the same hospital or to other medical facilities. In some cases, this response was late, resulting in hospitals and even entire cities (including Suceava County) entering a complete lockdown. Our staff was distributed to other departments in the hospital that were taking care of COVID-19 patients. For more than 2 months, all elective cases of Pediatric Surgery were canceled and the emergency cases were diverted to other close 2 hospitals. We resumed our regular activity on June 1, 2020; between this date and March 31, 2022 (beginning and end points of our study, respectively) a number of 1.598 patients were discharged from our department. This number represented a significant decrease compared to a similar time interval 2 years before (June 1, 2018–March 31, 2020), with 2.334 patients discharged (a decrease of 31.54%).^[55]^ We consider these changes to be mostly related to COVID-19 pandemic, similar to other reports worldwide. Although the incidence of SARS-CoV-2 positive children was small, the overall pediatric surgery activity was significantly decreased as part of the global challenge raised to the health system by the COVID-19 pandemic.

Our team made significant efforts to ensure the continuity of pediatric surgical activity during the COVID-19 pandemic by maximizing the use of available resources and limiting exposure to SARS-CoV-2 infection. The regular activity was covered by one pediatric surgeon working a 12-hours. shift; another pediatric surgeon was available for difficult cases such as trauma or complicated pathology (intestinal obstruction, ovarian or testicular torsion, etc.). The medical staff was redistributed such as to cover both COVID-19-positive and non-COVID-19 surgical areas by rotation, without interference between the 2 sectors. This task was accomplished with the participation of the Pediatric Department staff, in order to have enough personnel and room available for all pediatric patients. We encouraged early discharge after surgery in order to minimize the risk of virus transmission to non-COVID-19 patients and increase the availability of beds and healthcare personnel. In accordance with the hospital policy, we adapted our surgical protocols based on the availability of wards, Pediatric Intensive Care Units (PICU), ORs, and specialized personnel.

## 5. Conclusions

The incidence of SARS-CoV-2 positive surgical patients was small (around 1%) among children who were admitted to our Pediatric Surgery Department during the COVID-19 pandemic. There were only mild COVID-19 cases and no significant complications were recorded. However, the challenge raised by the COVID-19 on the health system also influenced the delivery of pediatric surgical care in terms of canceling elective procedures at the beginning of the pandemic. Proper adjustments included adequate training of the existing workforce, keeping the patients and medical staff safe, and maximizing the hospital resources’ utilization. A lesson we and others have learned is that a comprehensive strategy is needed by the public health authorities in order to avoid increased morbidity during a pandemic. This should also include a proper assessment comparison between the real risk of COVID-19 cross-infection and the benefits of elective procedures in areas such as Pediatric Surgery.

## Author contributions

**Conceptualization:** Florin Filip, Roxana Filip.

**Data curation:** Ramona Avramia, Monica Terteliu-Baitan, Maria - Elena Cocuz.

**Formal analysis:** Florin Filip, Roxana Filip.

**Investigation:** Florin Filip, Ramona Avramia, Roxana Filip.

**Methodology:** Florin Filip, Roxana Filip.

**Resources:** Florin Filip, Ramona Avramia, Monica Terteliu-Baitan.

**Software:** Florin Filip, Roxana Filip.

**Supervision:** Florin Filip.

**Validation:** Florin Filip, Ramona Avramia, Monica Terteliu-Baitan, Maria - Elena Cocuz, Roxana Filip.

**Writing – original draft:** Florin Filip, Maria - Elena Cocuz.

**Writing – review & editing:** Florin Filip, Roxana Filip.
